# Diagnostic Features for Human Categorisation of Adult and Child Faces

**DOI:** 10.3389/fpsyg.2021.775338

**Published:** 2021-11-19

**Authors:** Simon Faghel-Soubeyrand, Juliane A. Kloess, Frédéric Gosselin, Ian Charest, Jessica Woodhams

**Affiliations:** ^1^Département de Psychologie, Université de Montréal, Montréal, QC, Canada; ^2^School of Psychology, University of Birmingham, Birmingham, United Kingdom

**Keywords:** face age, facial feature, forensic psychology and legal issues, face perception and cognition, psychophysics, vision, spatial frequencies, orientation, indecent child images

## Abstract

Knowing how humans differentiate children from adults has useful implications in many areas of both forensic and cognitive psychology. Yet, how we extract age from faces has been surprisingly underexplored in both disciplines. Here, we used a novel data-driven experimental technique to objectively measure the facial features human observers use to categorise child and adult faces. Relying on more than 35,000 trials, we used a reverse correlation technique that enabled us to reveal how specific features which are known to be important in face-perception – position, spatial-frequency (SF), and orientation – are associated with accurate child and adult discrimination. This showed that human observers relied on evidence in the nasal bone and eyebrow area for accurate adult categorisation, while they relied on the eye and jawline area to accurately categorise child faces. For orientation structure, only facial information of vertical orientation was linked to face-adult categorisation, while features of horizontal and, to a lesser extent oblique orientations, were more diagnostic of a child face. Finally, we found that SF diagnosticity showed a U-shaped pattern for face-age categorisation, with information in low and high SFs being diagnostic of child faces, and mid SFs being diagnostic of adult faces. Through this first characterisation of the facial features of face-age categorisation, we show that important information found in psychophysical studies of face-perception in general (i.e., the eye area, horizontals, and mid-level SFs) is crucial to the practical context of face-age categorisation, and present data-driven procedures through which face-age classification training could be implemented for real-world challenges.

## Introduction

The amount of imagery depicting child sexual abuse (referred to here as indecent images of children, IIOC^1^; Edwards, 2013) in circulation has dramatically increased in the last 25 years, from estimates of thousands of such images in the late 1990s to millions or tens of millions nowadays (Home Office, 2017; Krasodomski-Jones et al., 2017). In the United Kingdom, the task of assessing whether a suspect’s digital material includes IIOC is conducted by digital forensics analysts who are members of specialist teams in police forces across the United Kingdom. This task involves determining whether a given image (a) depicts a child, and (b) if it is of an indecent nature (Kloess et al., 2019). In other countries, professionals undertaking the same task have the added challenge of distinguishing between children within specific age bands (e.g., in Germany, distinctions are made regarding IIOC that depict children under 18 years and children under 14 years; Ratnayake et al., 2014).

Identifying and classifying IIOC is a time-consuming task which is also stressful and emotionally challenging (Ratnayake et al., 2014; Franqueira et al., 2018). In addition, some imagery presents particular difficulties for the human classifier (Ratnayake et al., 2014; Kloess et al., 2019). Unsurprisingly, the potential to (semi-)automate the identification and classification of IIOC using algorithms is currently being explored (e.g., Gao and Ai, 2009; Sae-Bae et al., 2014; Home Office, 2017). Developing our understanding of what makes children’s faces distinct from adult faces, in particular the features used by human observers to discriminate them, is therefore an important step in developing software and advancing training for digital forensics analysts (and allied professionals). This study builds on existing qualitative research that has explored the aspects and attributes within imagery that digital forensics analysts report drawing on in order to inform their decision-making in the process of identifying and classifying IIOC, including specific facial and bodily features of children (Kloess et al., 2019, 2021; Michalski et al., 2019), by applying a new (data-driven) reverse-correlation technique relying on Gabor wavelets to this problem area. In doing so, it fills important gaps in the literature, given (1) the scarcity of studies that have examined facial differences associated with age (Gao and Ai, 2009) and, most importantly, (2) the absence of studies having revealed the specific facial features human observers use for age categorisation.

There are many physical changes associated with the maturation of faces. During childhood, craniofacial growth alters the shape and size of a child’s face, as well as the relative positions of different facial features. These changes are due to the development of permanent teeth and puberty. The greatest changes are seen between birth and the age of 5 years, with the rate of change being non-linear (Ricanek et al., 2015). Compared to adult faces, children’s faces are typically characterised by a “protruding forehead, large head, round face, big eyes and a small nose or mouth” (Komori and Nittono, 2013, p. 285).

Findings from research on the border-control task of the facial matching of children demonstrate that differentiating between faces of similar-aged children is difficult for non-expert (Kramer et al., 2018) and expert (i.e., nursery workers and super-recognizers; Belanova et al., 2018; Bate et al., 2020) observers, and that this task is harder the younger the child who is depicted (Michalski et al., 2019). In combination with research regarding how child and adult faces differ physically, this suggests that, particularly with younger children, their faces are defined by a common set of qualities which differ to the faces of adults. While these qualities may make the task of face-matching children more difficult, they should make the task of discriminating between child and adult faces easier, assuming that those who are engaged in the discrimination task attend to valid features for age categorisation.

In relation to this (Kloess et al., 2019), assessed the inter-rater agreement of law enforcement personnel experienced at identifying and classifying IIOC, finding levels of agreement that were not always adequate. In subsequent focus groups, the officers reported that features they found more indicative of a younger age included large eyes relative to the rest of the face, the presence of milk teeth, or the eruption of adult teeth.^2^ They also reported that the classification task was more challenging when there was a mismatch between the apparent maturity of the depicted person’s face and their body (or vice-versa), and where the depicted person was made to look younger or older (e.g., by means of make-up and clothing). More recently (Kloess et al., 2021), followed up on their 2019 study by asking digital forensics analysts to describe the key facial attributes they use in determining the presence of a child in imagery. These included: (1) large eyes, (2) a small nose, (3) a round facial shape, (4) an absence of cheekbones, (5) early signs of teeth development, and (6) smooth skin.

Attending to the face (rather than other cues) may thus improve the accuracy of the identification and classification of IIOC. It is therefore important to further investigate (1) whether participants do indeed rely on the features previously identified by police staff when discriminating between child and adult faces, and, relatedly, (2) whether attending to these features/facial areas does indeed lead to more accurate decision-making. In this matter, psychophysical studies on face perception have outlined the behavioural relevance of important features of facial images, such as orientation structure (Goffaux et al., 2016; Goffaux and Greenwood, 2016; Duncan et al., 2017), spatial location (Gosselin and Schyns, 2001; Adolphs et al., 2005; Faghel-Soubeyrand et al., 2019; Tardif et al., 2019), colour (Young et al., 2013; Benitez-Quiroz et al., 2018; Dupuis-Roy et al., 2019), and spatial frequency (SF) (Tardif et al., 2017; Estéphan et al., 2018; Faghel-Soubeyrand et al., 2020). However, while the use of these physical properties have been revealed for various face-processing tasks (e.g., face-identification, face-expression, and face-gender recognition in the above-cited studies), the diagnostic features for face-age recognition have never been explored.

The present study tackles this question by employing a recently developed reverse-correlation technique, called Diagnostic Feature Mapping (DFM; Alink and Charest, 2020), in order to precisely quantify the use of three core physical properties of facial images of children and adults, namely location, orientation structure, and SF. These properties are randomly sampled in a similar way to Bubbles (Gosselin and Schyns, 2001). A multiple linear regression applied to these samples and response accuracy can reveal the diagnosticity of behaviourally relevant features of images which are known to be crucial to the human visual system (i.e., different SFs, orientations, spatial coordinates, and sizes in the striate cortex; Hubel and Wiesel, 1968). Contrasting with previous psychophysical studies, however, is the capacity of DFM to reveal these three core features in a single experimental paradigm, permitting a thorough description of the facial information relied for categorisation tasks. Using such Gabor features was also motivated by studies in computer vision (Gao and Ai, 2009), suggesting that models using Gabors as priors perform well in explaining differences between facial images of children and adults. DFM therefore provided an informed, comprehensive and entirely data-driven way to reveal the specific facial features associated with face-age categorisation.

## Method

### Participants

A screening questionnaire was used to screen for conditions that would prevent a volunteer being able to take part in the experiment. This resulted in one volunteer being excluded. Of the 16 participants who took part in the experiment (male = 9, female = 7, right-handed = 14, left-handed = 2, White British = 12, and Asian-Indian = 4, aged between 21 and 47 years (*M* = 38.44, SD = 8.33), seven were students or staff at the University of Birmingham, and nine were digital forensics analysts recruited from West Midlands Police. All participants had no history of psychiatric diagnosis and all had normal or corrected-to-normal vision. Full ethical approval for the study was granted by the Science, Technology, Engineering and Mathematics Ethical Review Committee at the University of Birmingham (ERN_15-1374AP10A). In addition, the researchers adhered to the British Psychological Society’s Code of Ethics and Conduct (2018) throughout the study, and the experiment was conducted in accordance with the Declaration of Helsinki. Participants were recruited through the University of Birmingham’s Research Participation Scheme, *via* the last author’s professional network at the University of Birmingham, and by means of an advertisement that was circulated *via* internal channels within West Midlands Police to officers who were working with child sexual exploitation materials on a daily basis. This study was not preregistered.

### Stimuli

A total of 12 images depicting faces of either children or adults (six adults, six males; all with neutral expressions) were selected from the Radboud Face dataset (Langner et al., 2010), and converted into 250 × 250 pixels greyscale images. Each image was aligned based on 20 landmarks (averaged to six mean coordinates for left and right eyes, left and right eyebrows, nose, and mouth) using Procrustes transformations (rotation, scaling, and translation), and revealed through an elliptical mask that excluded non-facial cues such as hair, ears, and neck. The luminance profile of the resulting face images was equalised across images using the SHINE toolbox (Willenbockel et al., 2010).

### Gabors Wavelet Decomposition

**FIGURE 1:**
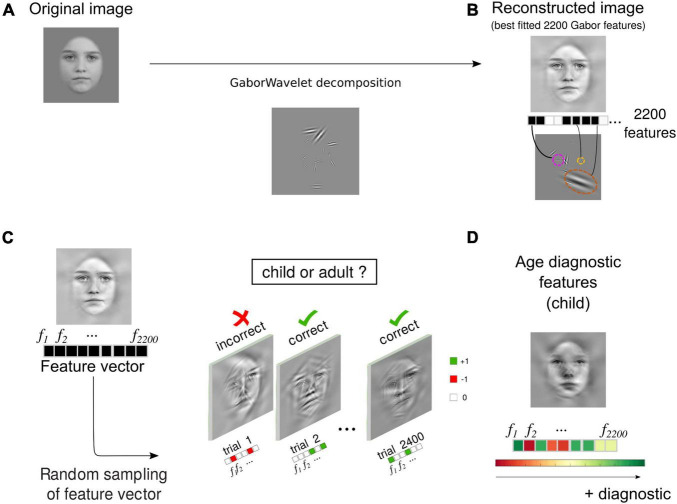
Image feature decomposition and reverse-correlation procedure. A reconstructed image (**B**, lower image) was created using a subset of 2200 Gabors wavelets varying in spatial-frequency (from 2.4 to 87 cpi), orientation (10°–180°), *x*–*y* coordinates, and size. These 2200 wavelets, the most correlated with the original image **(A)**, are referred to as Gabor features (see also Alink and Charest, 2020 for further details). **(C)** At any given trial, the stimulus was created by randomly sampling the 2200 Gabor features to reveal parts of the reconstructed image. Participants were asked to indicate whether the resulting sampled face was a child or an adult. Each participant was submitted to 2.4k trials, for a grand total of >38k trials. **(D)** We obtained the diagnostic features by weighing and summing the randomly sampled feature vectors across trials with *z*-scored behavioural accuracies. (Image source: Langner et al., 2010).

A more in-depth description of the Gabors wavelet decomposition algorithm has been published elsewhere (Alink and Charest, 2020). Briefly, we used a custom-made MATLAB program which aimed to reduce each face image to a subset of the most important 2200 Gabor wavelets. To do so, we considered wavelets of 20 different SFs, exponentially increasing between 2.4 and 87 cycles per image ($SF=\frac{10}{45}\times 1.08^{n};n=\left[1,2,\ldots ,20\right]$). They had 12 orientations between 15° and 180° in constant steps of 15°, and were centred on each possible pixel of the input greyscale image. The final sets of Gabor features (Figure 1B) were selected based on their best fit (least-square correlation) to the original greyscale images (Figure 1A). Amplitudes were set to an equal value for all wavelets. Partial reconstructions of the images at every given trial were created by randomly selecting a subset of the 2200 Gabor wavelets, and summating them (see Figure 1C). The resulting range of pixel values was modified linearly so that all stimuli covered the full 0–255 range.

### Apparatus

The experimental program ran on Windows computers in the MATLAB environment (The MathWorks, Natick, MA, United States), using functions from the Psychophysics Toolbox (Watson and Pelli, 1983; Brainard, 1997). Stimuli were shown on 22-inches monitors (1920 × 1080 pixels at 60 Hz). Participants performed the experiment in a dimly lit room, and viewing distance was maintained at ∼76 cm.

### Experimental Procedure

The experiment took place in the laboratories of the School of Psychology at the University of Birmingham. Participants were first given standardised instructions about the experimental task. Each trial began with a grey screen and a central fixation point (250 ms), which participants were instructed to gaze at. The partial face reconstruction spanning 8° of visual angle was then shown in the central area of the screen, and remained visible until the participant’s response. Participants were asked to categorise the faces depicted in the stimuli as either children or adults as fast and as accurately as possible. The stimulus presentation order was randomised for each participant. All 16 participants completed 2400 trials of this adult vs. child face discrimination task, with short breaks every 200 trials (approximately 10 min). The task was completed in one session that lasted between 1.5 and 2 h. Overall, a total of 38,400 trials were recorded for this study. Estimates of effect size could not be assessed considering the lack of previous studies on face-age recognition, but the current number of observations per participant is more than twice the typical range used in reverse-correlation studies (e.g., Tardif et al., 2019).

The quantity of face information (i.e., the number of Gabor Wavelet features) necessary to maintain an accuracy of 75% was adjusted on a trial-by-trial basis with the QUEST algorithm (Watson and Pelli, 1983). Royer et al. (2015) showed that such a threshold (measured using a similar method) is strongly correlated with three commonly used face recognition ability tests (*r* = −0.79 with the mean of the Cambridge Face Memory Test +, the Cambridge Face Perception Test, and the Glasgow Face Matching Test short version). We thus used the number of Gabors required to maintain an accuracy of 75% as our individual index of face-age discrimination performance.

After the experiment, participants were verbally debriefed and were given a debriefing sheet to take with them. Participants recruited through the Research Participation Scheme were compensated with course credits and participants from West Midlands Police were reimbursed for their travel expenses and time.

### Data and Code Availability

The data and MATLAB code that support the findings of this study are available from the corresponding author upon reasonable request.

### Feature Diagnosticity Index

We quantified how the Gabor features (the Gabor wavelets) were differently associated with face representations by computing a Feature Diagnosticity index (FDi; see Alink and Charest, 2020) for each of the features composing our face images, for every participant, and stimulus independently (see Figure 1D). This was done by weighing the features presented across trials by the *z*-scored accuracy values, providing us with a three-dimensional matrix of FDi with the dimensions number of participants (*n* = 16), number of images (12), and number of features (2200).

## Results

Accuracy was close to the target 75% (*M*_*acc*_ = 0.7577, SD_*acc*_ = 0.0443). One participant was excluded from further analysis because of poor performance (number of Gabors threshold was greater than 3 SD above the mean of all participants). The average number of Gabors required to attain 75% accuracy ranged from a low of 156.52 (i.e., best performance) to a high of 443.75 (i.e., lowest performance; *M*_*nbgabors*_ = 275.52, SD_*nbgabors*_ = 73.86).

### Facial Areas for Child and Adult Classification

**FIGURE 2:**
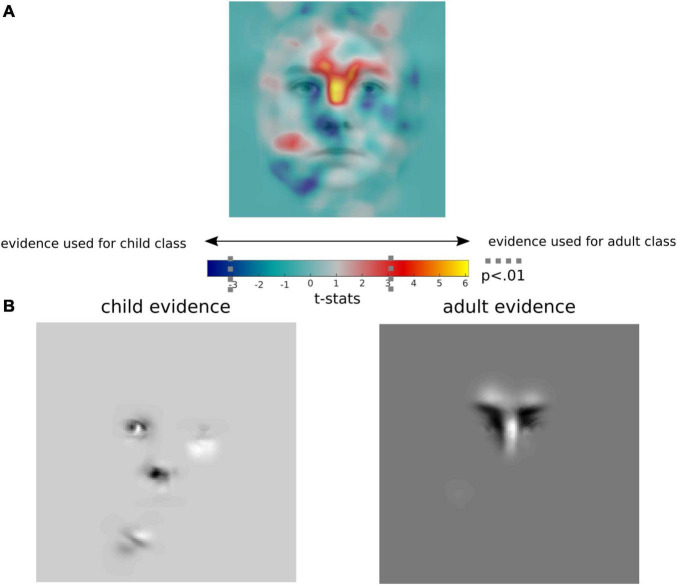
Facial features for child and adult categorisation. Regression coefficients for 2D feature positions (for accurate classification of child and adult images) were spatially smoothed, and compared between child and adult faces with unpaired *t*-tests. On the first panel **(A)**, hot colours (positive *t*-statistics) indicate evidence for accurate adult classification, and cold colours (negative values) indicate evidence for accurate child classification. The two bottom panels **(B)** show pixels (i.e., regions of faces) significantly linked to accurate child and adult categorisation, respectively. (Image source: Langner et al., 2010).

To reveal the facial attributes used to categorise faces as child vs. adult, we reprojected each Gabors FDi to their specific *x*–*y* image position, smoothed (with a Gaussian kernel with a standard deviation of 10 pixels) the resulting image regression coefficients for each stimulus, and compared them between children and adults faces with unpaired *t*-tests. These comparisons of regression coefficients are displayed on Figure 2A, with hot colours (positive *t*-statistics) indicating evidence for accurate adult classification, and cold colours (negative values) indicating child classification evidence. Significant facial features (*p*s < 0.05) for adult classification evidence were found in an area around the nasal bone and eyebrows structure. In contrast, we found that the eyes, nose, and jawline were used as evidence for child face categorisation. The significant regions have been highlighted on an average of all face stimuli on Figure 2B for child evidence (left bottom image) and adult evidence (right bottom image). The average regression coefficient image (irrespective of category type) is shown in Supplementary Figure 1A.

### Spatial-Frequencies for Child and Adult Classification

**FIGURE 3:**
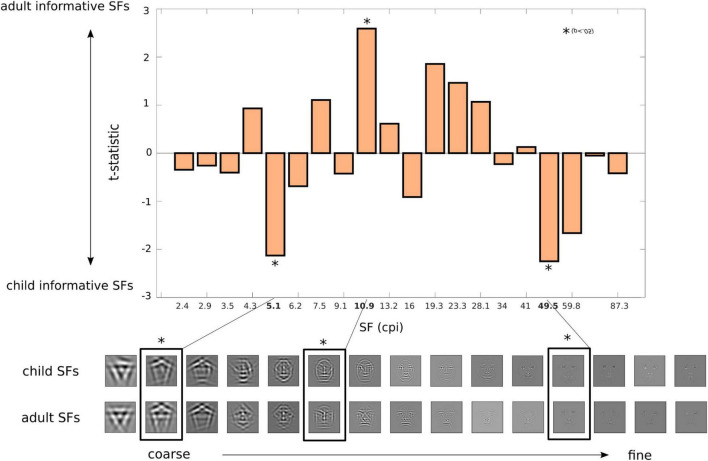
Spatial frequency evidence for categorisation of child and adult faces. Correlation coefficients for feature granularity (for accurate classification of child and adult images) were compared between child and adult faces for each one of the 20 SF bins (2.4–87.3 cpi) with unpaired *t*-tests. For visualisation purposes, the lower panel images display the facial information for each of the SF bands for child and adult faces. Here, positive *t*-statistics shows SF evidence for child-like faces, while negative values shows SF evidence for adult-like faces. Significant SF bands – low SF of 5 cpi and high SF of 50 cpi for child faces, mid-SF of 22 cpi for adult faces – are outlined with an asterisk.

To determine the SFs (or information granularity) used to accurately categorise child and adult faces, we correlated the SF parameters of the presented Gabor features with accurate responses for child and adult faces across trials for all participants. The regression coefficients were compared between child and adult faces with unpaired *t*-tests. The resulting evidence magnitude for the 20 SFs bands are shown in Figure 3, with significant SF bands (*t*-stats with *p* < 0.05) highlighted. Overall, this revealed that low (coarse details of 5.1 cpi) and high SFs (fine details of 49.5 cpi) were used as evidence of child faces, while mid-SFs were relied on by our participants as evidence for adult classification. The average SFs regression coefficient (irrespective of category type) are shown in Supplementary Figure 1B.

### Orientation of Facial Traits for Child and Adult Classification

**FIGURE 4:**
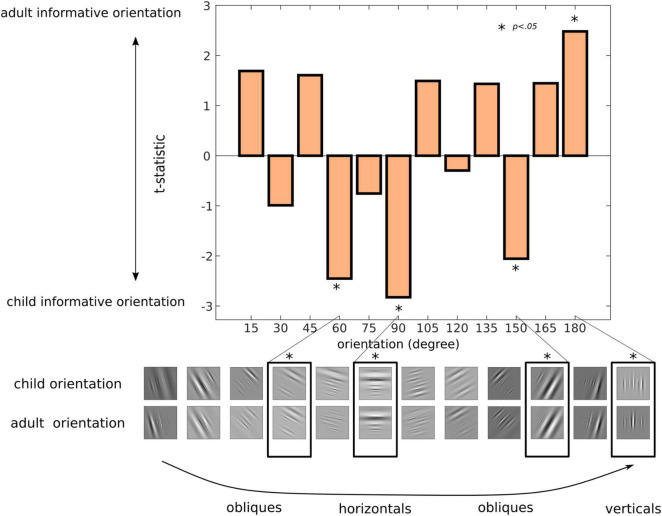
What orientation structure do we rely on to categorise child and adult faces? Correlations coefficients for feature orientation (for accurate classification of child and adult images) were compared between child and adult faces for each one of the 12 orientation bands (10°–180° in 15° steps) with unpaired *t*-tests. For visualisation purposes, the lower panel images display the facial information for each of the 12 orientation bands for child and adult faces. Positive *t*-statistics shows orientation evidence for child-like faces, while negative values shows orientation evidence for adult-like faces. Significant orientation bands – horizontal (90°) and oblique (60° and 150°) information for child faces, vertical information (180°) for adult faces – are outlined with an asterisk.

To determine the type of orientation structure relied upon for accurate categorisation of child and adult faces, we correlated the orientation parameter of the presented Gabor features with accurate responses for child and adult faces across trials for all participants. The regression coefficients were then compared between child and adult faces with unpaired *t*-tests. The corresponding *t*-statistics are shown in Figure 4, with significant orientation bands (*t*-stats with *p* < 0.05) highlighted. Overall, this revealed that horizontal (90°) and oblique facial structures (60° and 150°) were used as evidence for classification of a face as a child. For adult face categorisation, however, vertical features (180°) were linked to accurate responses in our participants. The average orientation regression coefficient (irrespective of category type) is shown in Supplementary Figure 1A.

## Discussion

What specific visual features drive our accurate categorisation of faces as either a child or an adult? One practical implication of answering this question is the improvement of the identification and classification of IIOC (Edwards, 2013) which involves determining whether a given image (a) depicts a child, and (b) if it is of an indecent nature (Home Office, 2017; Kloess et al., 2019). Here, we used a data-driven approach – DFM (Alink and Charest, 2020; see also Gosselin and Schyns, 2001 for a similar approach) to reveal the specific facial features human participants draw on in order to distinguish child vs. adult faces. Specifically, we revealed the use of three types of features important to the visual system – SF, position, and orientation – that human observers extract while they complete child vs. adult categorisations, yielding a precise and comprehensive characterisation of the relevant facial information for face-age categorisation.

The participants in the present study used information located around the nasal bone and eyebrows (supraorbital ridge) to classify adult faces. There was some evidence that they also used an area of the cheek, which may correspond with the flattening of the cheeks that occurs during the transition from childhood to adulthood (Kau and Richmond, 2008). In contrast, the area around the eyes, nose, and jawline were more indicative of a child’s face. Indeed, this matches with physical differences in faces of children: large eyes relative to the rest of the face, a small, wider, up-turned nose with a concave bridge, and a rounder chin, are all physiological characteristics that have been reported (Liggett, 1975; Sforza et al., 2011). Our findings are also in line with some of the facial features reported by digital forensics analysts in Kloess et al. (2021), who explicitly described the following facial attributes as indicative of a younger age: (1) large eyes, (2) a small nose, (3) a round facial shape, and (4) an absence of cheekbones. The present study corroborated these findings by revealing, with implicit and data-driven methods, the facial attributes linked to accurate child vs. adult categorisation. But our DFM paradigm enabled us to go further than revealing facial areas for accurate face-age categorisation from faces.

Indeed, DFM’s reliance on Gabor wavelets parameters enabled us to show the specific level of detail (SFs) and orientation structure humans extract from images of faces in order to determine the age from a face. We found that the SFs associated with age classification manifested a U pattern from low to high SF, with low SF (coarse features) and high SF (fine features) being associated with child faces, and mid-level SF being associated with adult faces. The fact that coarse facial features are associated with younger, child-like faces aligns with the layman notion that some facial features of children are larger, such as the forehead, the head, and the eyes (Komori and Nittono, 2013). Interestingly, the mid-level SFs (10 cpi) found to be indicative of adult faces matches the level of information required for (adult) facial identification found in previous studies (e.g., Willenbockel et al., 2010; Tardif et al., 2017, 2019). This might explain, in turn, why identification of children’s faces has been reported to be so difficult in lab conditions (Belanova et al., 2018; Kramer et al., 2018; Bate et al., 2020), as well as in real-world conditions (e.g., Kloess et al., 2019, 2021); the level of detail of facial information for our classification of a face as a child (i.e., low and high-SFs) does not match the facial information we normally use for adult face-identification (i.e., mid-SFs).

For orientation information, we found that vertical features of a face, presumably around the nasal bone, is indicative of an adult’s face. Indeed, as shown in Figure 2, the eyebrow region as well as the nasal bone were associated with accurate identification of stimuli as an adult. These features coincide with areas of the skull that reliably differentiate the sexes in adult skulls, namely the glabella and the supraorbital ridge (Tanner and Tanner, 1990; Graw et al., 1999). Between puberty and adulthood, men develop a prominent supraorbital ridge and a larger glabella; in younger males, these features are not as apparent leading to uncertainty when classifying younger skulls as male or female (Graw et al., 1999). Our participants may therefore be using features reliably associated with adult male faces to differentiate child from adult faces in our sample.

In contrast, oblique features (i.e., specifically information at angles of 60° and 150°) were more indicative of a child’s face, presumably from the rounder jawline and eyes of children’s faces. In addition, we found that horizontal information – which is paramount for face identification and the categorisation of facial expressions of emotions (Goffaux and Dakin, 2010; Pachai et al., 2013; Goffaux et al., 2016; Goffaux and Greenwood, 2016; Duncan et al., 2017) – was significantly associated with child categorisation. In fact, information for face-age categorisation peaked around this horizontal band (specifically at 75° when averaged across both child and adults). This finding is yet another piece of evidence showing that horizontal information is at the core of face processing tasks, and extends the importance of this information for the first time to the challenging task of face-age categorisation. Our findings also go a step further than previous psychophysical studies of face perception by showing that it is the horizontal information in the eye region that is the most relevant in the context of this face processing task. To our knowledge, this is the first time horizontal information is linked in a direct manner to eye information in a face processing task (e.g., see Duncan et al., 2017 for an indirect link).

In terms of the real-world implications of our findings, there appears to be a common set of facial features that is associated with children’s faces, which may explain why differentiating between faces of different children can present such a challenge (e.g., the task facing border control agents; Belanova et al., 2018; Kramer et al., 2018; Michalski et al., 2019; Bate et al., 2020). Our findings are also relevant to the task of identifying IIOC, whether by a human or an AI algorithm, and provides an evidence base for the training of digital forensics analysts (and applied professionals) who have to differentiate between faces of children from those of adults. It is reassuring that our findings coincide with some of the features described in accounts given by experts in this task as to the features to which they attend (Kloess et al., 2021). However, by incorporating our findings into training, this knowledge can be imparted to new recruits, ensuring that they are using the features empirically demonstrated to be associated with accurate differentiation from the start. Our findings are also relevant to discussions about verifying children online for safeguarding (GCHQ, 2020), and to technology companies who design age assurance algorithms that interface with a webcam (e.g., Yoti).

Finally, it is important to acknowledge the limitations of our study. The facial stimuli represented 12 individuals and all were of a White ethnicity. Three key implications stem from this: (1) our method needs replication with a larger set of faces; (2) it should be extended to the study of faces of other ethnicities (Gao and Ai, 2009); and (3) the interaction between the ethnicity of the viewer and the ethnicity of the stimuli should be evaluated. According to the Global Threat Assessment completed by the We Protect Global Alliance (2019), children from lower income countries are at particular risk of sexual exploitation and abuse in order to provide family income given the high level of poverty in some of these areas. This also results in an increase of non-White children being depicted in IIOC, which the analysts in the Kloess et al. (2021) study reported as presenting an additional challenge due to the lack of familiarity with those of a different ethnicity to their own, and the fact that different ethnicities can represent great variation in terms of the stages and nature of physical development.

In summary, we used a comprehensive data-driven technique to reveal how three major types of facial information are used by human observers to make face-age categorisations. Notably, the sampling of all three visual features – position, SF, orientation – simultaneously in the DFM procedure permitted us to interpret and integrate these findings in a single experiment/data set, relating spatial information (e.g., features of the jawline) with orientation information (e.g., oblique information). The features of the face our participants relied on to differentiate child from adult faces align with what is known about changes in facial physiology from childhood to adulthood, and with some of what digital forensics analysts have self-reported about the features to which they attend when trying to classify IIOC. These results are the first to show the *relevance* of specific facial features for age classification by providing evidence that attending to these features (over others) is associated with *accurate* face-age classification.

## Conclusion

In this study, we employed reverse correlation to reveal the key facial features used by human observers for face-age categorisation. The diagnostic image features that we identified correspond to core psychophysical features of human face perception (i.e., features in the eyebrow and nose area, those in the mid SFs, and those around the horizontals). This not only advances our knowledge of the psychophysical correlates for face-age categorisation for the first time, but also constitutes a crucial step toward implementing evidence-based face-age classification training. Indeed, the features we identified could be used to inform current efforts to identify child vs. adult faces, be this by human or machine (e.g., identifying and classifying IIOC or for age verification purposes). We have already shown, for example, that inducing the use of the best set of features in human observers is fruitful in enhancing their performance for other face classification tasks (Faghel-Soubeyrand et al., 2019). Police-analysts could thus be trained to focus their attention on the features outlined here to enhance their performance in detecting a child in IIOC. Another interesting application of our findings would be to build better automatic (computer) algorithms for child vs. adult face classification by biasing the weights of pattern recognition algorithms [i.e., in Deep Convolutional Neural Networks, such as the ones used in Qawaqneh et al. (2017)] toward the useful set of features revealed in this study. Indeed, features humans use are generally more robust for image classification than those used by machine learning algorithms (Geirhos et al., 2018). However, we think that any such further developments will require the inclusion and representation of stimuli and participants from ethnic minority groups, as well as individuals with strong perceptual skills such as super-recognizers. These additions, we believe, will be important steps toward our findings being generalizable, as well as developing high-performing classification algorithms and experimental tools that support forensic and legal decision-making more generally.

## Public Significance Statement

Understanding how we recognise children from adults has wide-reaching applications, such as the improvement of the classification of indecent images of children by police analysts and computer vision algorithms. Here we show a thorough characterisation of the visual strategies human observers use to recognise children from adults, providing data-driven associations between specific facial features and human accurate age classification.

## Data Availability Statement

The data and MATLAB code that support the findings of this study are available from the corresponding author upon reasonable request.

## Ethics Statement

The studies involving human participants were reviewed and approved by the Science, Technology, Engineering and Mathematics Ethical Review Committee at the University of Birmingham. The patients/participants provided their written informed consent to participate in this study.

## Author Contributions

SF-S and IC conceptualised and programmed the experimental procedure. JW and JK helped in the recruitment and testing of the participants. SF-S completed the analysis scripts as well as the figures of the manuscript. SF-S, JK, and JW wrote the first draft of the manuscript. FG, JK, IC, JW, and SF-S revised the versions of the manuscript. All authors contributed to the article and approved the submitted version.

## Author Disclaimer

The authors would like to express their gratitude and appreciation to West Midlands Police for their assistance, time and effort in supporting the study undertaken and presented here, as well as the Institute for Global Innovation for financially supporting this study.

## Conflict of Interest

The authors declare that the research was conducted in the absence of any commercial or financial relationships that could be construed as a potential conflict of interest.

## Publisher’s Note

All claims expressed in this article are solely those of the authors and do not necessarily represent those of their affiliated organizations, or those of the publisher, the editors and the reviewers. Any product that may be evaluated in this article, or claim that may be made by its manufacturer, is not guaranteed or endorsed by the publisher.

## Footnotes

1 This material is also referred to as child sexual exploitation material (Merdian et al., 2021), child pornography, and sexually exploitative material of children (Franqueira et al., 2018).

2 The use of dentition developmental stage in making accurate age assessments has been questioned, and may therefore only be useful in images where the depicted person’s mouth is open (see Ratnayake et al., 2014), which is not always the case in the context of IIOC.
