# Supplementary material for: Diagnostic Features for Human Categorisation of Adult and Child Faces
Source: Front Psychol. 2021 Nov 19;12:775338. doi: 10.3389/fpsyg.2021.775338 (PMC8640236; doi:10.3389/fpsyg.2021.775338)
Supplement: Supplementary file 1 [file Data_Sheet_1.pdf]

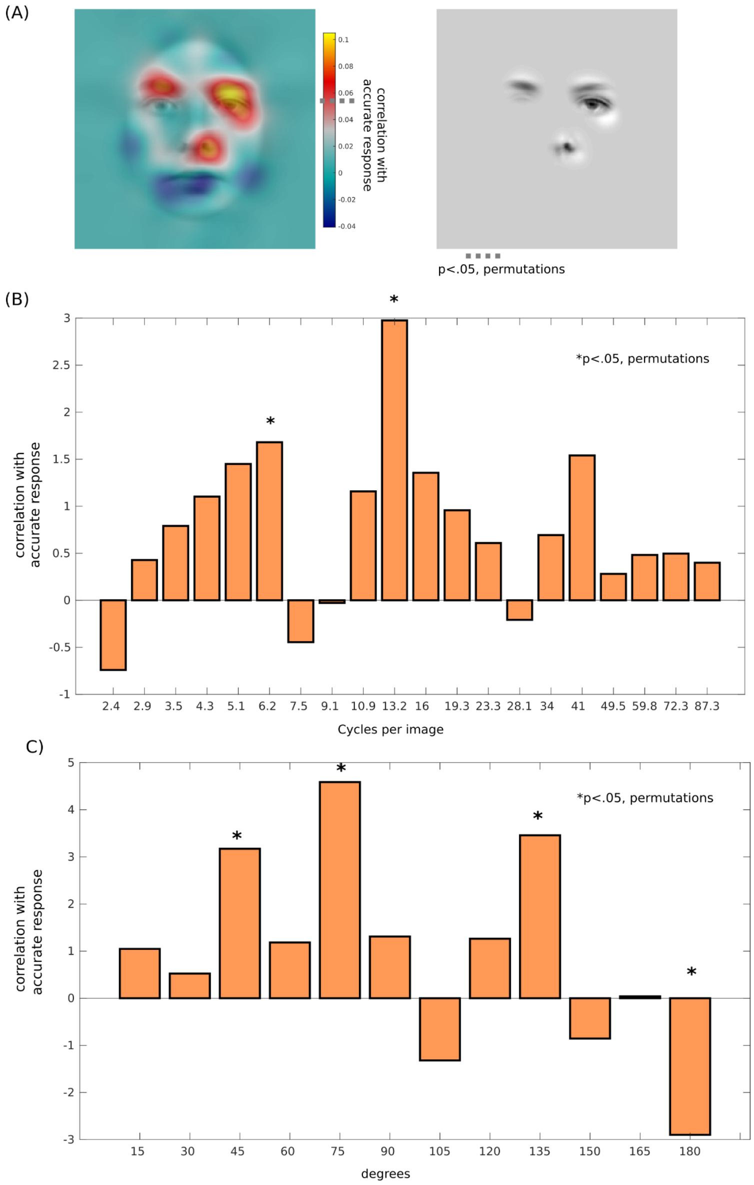

supplementary figure 1. Features for accurate classification of child vs adult faces, irrespective of the class. Correlation between accurate responses and position (A), spatial-frequencies (B) and orientations (C) have been averaged irrespective of the stimulus class. Significance was assessed using permutation testing : a null distribution was created by regressing the presence of each feature type across trials with randomly shuffled the accuracy vector of each participant (repeated 1000 times).
